# Desert mammal populations are limited by introduced predators rather than future climate change

**DOI:** 10.1098/rsos.170384

**Published:** 2017-11-01

**Authors:** Aaron C. Greenville, Glenda M. Wardle, Chris R. Dickman

**Affiliations:** 1 Desert Ecology Research Group, School of Life and Environmental Sciences, University of Sydney, Sydney, Australia; 2 Long Term Ecological Research Network, Terrestrial Ecosystem Research Network, St Lucia, Australia

**Keywords:** climate change, wildfire, rainfall, bottom-up, top-down, predation

## Abstract

Climate change is predicted to place up to one in six species at risk of extinction in coming decades, but extinction probability is likely to be influenced further by biotic interactions such as predation. We use structural equation modelling to integrate results from remote camera trapping and long-term (17–22 years) regional-scale (8000 km^2^) datasets on vegetation and small vertebrates (greater than 38 880 captures) to explore how biotic processes and two key abiotic drivers influence the structure of a diverse assemblage of desert biota in central Australia. We use our models to predict how changes in rainfall and wildfire are likely to influence the cover and productivity of the dominant vegetation and the impacts of predators on their primary rodent prey over a 100-year timeframe. Our results show that, while vegetation cover may decline due to climate change, the strongest negative effect on prey populations in this desert system is top-down suppression from introduced predators.

## 1. Introduction

Interactions among species are crucial for ecosystem functioning and for maintaining biological diversity [1,2]. They drive the dynamics of species populations and key processes such as nutrient cycling, and facilitate plant–pollinator networks and predator–prey relationships. Interactions can occur locally between pairs of species, but also may exhibit complex nonlinear dynamics across multiple scales [3]. Importantly, species interactions can be modified by climate [4,5], with recent work suggesting that one species in six is at risk of extinction due to global climate change [6]. To date, most studies have investigated non-interactive effects of climate change across trophic levels and focus on single or independent multiple processes [7], or explore the ecology of individual species or groups of species using habitat or niche modelling and dispersal capacities. Biotic interactions are usually ignored [8], even though such interactions can profoundly alter how biota respond to climate change (e.g. [9]). These observations make it imperative for ecologists to investigate how the interactive effects of multiple population processes affect the diversity and functioning of ecosystems [10,11], both now and in future.

Climate change has the potential to increase or decrease the strength of interactions and the effects of other ecological processes. Climate change may have different effects if the interactions or processes are positive or negative, leading to unpredictable changes in the composition of species assemblages. Identifying the importance of particular biotic interactions and other processes within broader networks is difficult, particularly when multiple trophic levels are involved, but can be useful for detecting the first evidence of environmental change [12]. For example, small shifts in plant phenology may have large effects on mutualisms, such as plant–pollination networks [13]. Extreme weather events, such as droughts, can disrupt flowering phenology, leading to declines or even local extinctions of pollinators, which in turn can have cascading effects on frugivores and seed dispersal [14,15].

Antagonistic interactions may also intensify under a changing climate [5,7]. For example, a review of 688 studies found that competitive interactions between plants were consistently shifting due to climate change [7]. However, predator–prey interactions were affected negatively or positively [7], highlighting both the difficulties of reliably predicting species' population changes and the need for studies of individual systems.

Climate also plays a key role in driving other abiotic processes that influence species populations, as exemplified by the pulse-reserve paradigm that applies in many arid environments [16]. The pulse-reserve paradigm states that deserts are water-limited and predicts that consumer populations should respond positively to pulses of rainfall through bottom-up processes [16]. For example, infrequent rainfall events can lead to pulses in primary production and flow-on to increases in consumer populations that structure both populations and assemblages [17]. More recently, research in arid regions has shown that these environments can exhibit both bottom-up and top-down control of species populations, and climate conditions frequently alter the timing and intensity of trophic connections [18].

Pulses of productivity from global climate cycles also increase fuel loads. For example, the El Niño-Southern Oscillation cycle drives wildfires across the Americas and Oceania [19] that have pervasive effects on biological diversity [20]. In central Australia, risk of wildfire increases after large, yet rare, rainfall events that promote high fuel loads [21–23]; such events are currently increasing in frequency and magnitude [24]. Temperatures, and hence evaporation rates, in central Australia have also increased over the last 100 years, further exacerbating the likelihood of more frequent and intense wildfires [25]. In addition to changes in species interactions, such dramatic climate shifts are likely to accelerate the global risks of extinction for many species and ecosystems. Recent analyses suggest that these risks are greater for South America and Australia than for other continental regions [6], with many taxa in Australia's central arid regions being particularly vulnerable [26].

Phenomenological models provide a useful framework for modelling ecological interactions and processes, and thus can help to improve our understanding of complex ecological systems [27,28]. Within phenomenological models, researchers can use a combination of existing theory, such as the pulse-reserve paradigm [16], and data to predict species' responses to climate change. While the pulse-reserve paradigm predicts rain-driven increases in productivity and consumer populations, top-down effects from predators may limit the population growth of consumers through predation and also reduce population growth among themselves via interference competition [29]. In general, introduced predators are predicted to cause population declines of consumers due to the usually elevated effects of their predation on native fauna [30,31]. Climate shifts that modify the pulse-reserve system can be expected to have flow-on effects that will further influence biota via both bottom-up and top-down processes. In semi-arid grassland communities in central California, for example, increased rainfall has been found to alter the strength of both consumer-resource and facilitative interactions, even reversing the direction of effects in some cases [32].

Here, we explore how species in multiple trophic levels interact with each other and with dynamically varying environmental drivers in a case study arid system, and investigate how stresses from global climate change may modify already-complex interaction networks. We first construct a conceptual phenomenological model based on central Australian biota that captures the key interactions and processes that operate there, and then apply structural equation modelling (SEM) using actual data on these biota to quantify how they are affected by key system processes. We base our phenomenological model on the pulse-reserve paradigm and the interactions derived by top-down forcing from predators. We parametrize the model using long-term (17–22 year) regional-scale datasets on rainfall, vegetation cover, time-since-wildfire, captures of small mammals and reptiles, and a shorter-term (2 year) dataset on both a native mammalian top predator and two species of smaller introduced predators. We then use the results from the structural equation model to make the following predictions, in line with future climate projections for central Australia [24]:

1. Under the pulse-reserve paradigm, increases in the frequency of heavy rainfall events will not compensate for increases in wildfire frequency, in turn decreasing both seeding events in the dominant vegetation (spinifex) and numbers of seed-eating rodents.
2. Removal of mammalian predators will lead to increased prey numbers due to the alleviation of top-down suppression, increased survival and reproduction, with the greatest increases in prey numbers following the removal of introduced predators.

The study region has a depleted but still-diverse assemblage of mammals and highly diverse assemblages of reptiles [22]; these taxa and key components of the vegetation, such as the dominant species or critical habitat for small vertebrates, are likely to be vulnerable to climate change [33].

## 2. Material and methods

### 2.1. Study site

**Figure 1.**
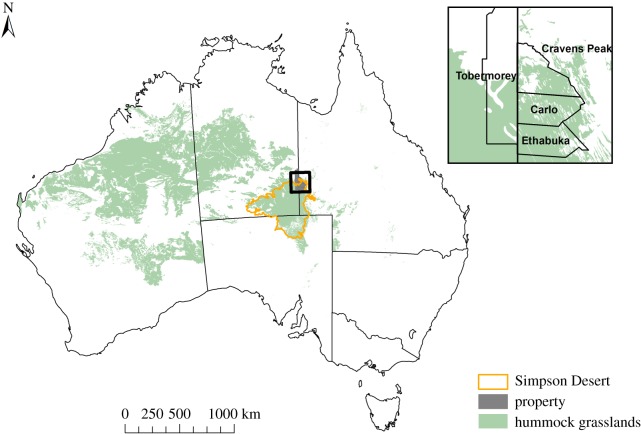
The location of the study region in the Simpson Desert, Australia. Insert shows location of properties where long-term vegetation, small mammal and reptile and mammalian predator monitoring occurs.

The study was conducted in the Simpson Desert, central Australia (figure 1). This region occupies 170 000 km^2^; dune fields comprise 73% of the region, with smaller areas consisting of clay pans, rocky outcrops and gibber flats [34]. The sand dunes run parallel in a north–south direction aligned with the prevailing southerly wind. The dunes are up to 10 m high and 0.6–1 km apart [35]. Vegetation in the interdune swales and on dune sides is predominantly spinifex grassland (*Triodia basedowii*) with patchy cover of small stands of gidgee trees (*Acacia georginae*) and other woody *Acacia* shrubs or mallee eucalypts; low-lying clay pans fill with water temporarily after heavy rain [33].

During summer, daily temperatures usually exceed 40°C and minima in winter often fall below 5°C [35]. Highest rainfall occurs in summer, but heavy rains can fall locally or regionally throughout the year. Long-term weather stations in the study area are at Glenormiston (1890–2011), Boulia (1888–2011) and Birdsville (1954–2011), and have median annual rainfalls of 186 mm (*n* = 122 years), 216.2 mm (*n* = 124 years) and 153.1 mm (*n* = 58 years), respectively [36].

### 2.2. Small mammal and reptile captures

We carried out live-trapping at nine sites across Carlo Station, Tobermorey Station, Cravens Peak and Ethabuka Reserves, an 8000 km^2^ region in the Simpson Desert, southwestern Queensland, Australia (figure 1). Small mammals (native rodents, *Notomys alexis, Pseudomys hermannsburgensis* and *Rattus villosissimus*, and dasyurid marsupials, including *Sminthopsis youngsoni*, *Sminthopsis hirtipes* and the predatory mulgara *Dasycercus blythi*) and reptiles were live-trapped using pitfall traps (16 cm diameter PVC pipe dug 60 cm deep into the ground), each equipped with a 5 m drift fence of aluminium flywire to increase trap efficiency [37]. Pitfall traps were arranged on 1 ha grids comprising six lines of six pitfall traps spaced 20 m apart (i.e. 36 pitfall traps per grid). Sites contained 2--12 grids and were spaced at least 10 km apart. Grids within sites were set 0.5–2 km apart in randomly chosen positions along access tracks. We opened traps two to six times a year from 1990 to 2012 for two to six nights at one site (Main Camp), and from 1995 to 2012 at eight more sites (Shitty Site, Field River South, Field River North, South Site, Kunnamuka Swamp East, Carlo, Tobermorey East and Tobermorey West). We conducted trapping sessions in both the Austral summer and winter each year to maximize species captures due to seasonal changes, such as rainfall and temperature. Each animal was given a unique individual identification mark. Long-term (17–22 years; up to 130 sampling trips per site) live-trapping (205 524 trap nights) yielded 21 202 captures of 11 species of small mammals and 17 681 captures of 58 species of reptiles [38]. We pooled animal captures per grid at each site per trip (sampling period; electronic supplementary material, table S1) and calculated the minimum numbers of animals known to be alive (i.e. total captures per grid–recaptures). Appropriate permissions and licences to conduct the fieldwork were obtained from the Queensland Government (Permits WITK15192514 and WISP15192514).

### 2.3. Predator monitoring

We monitored the activity of a top predator, the dingo (*Canis dingo*), and two species of smaller predator (or ‘mesopredator’), the introduced feral cat (*Felis catus*) and red fox (*Vulpes vulpes*), at each site as described in Greenville *et al.* [39]. In brief, 25 remote cameras were in continuous operation from April 2010 to April 2012 and adequately surveyed the three predator populations across both a resource pulse (‘boom’) and subsequent ‘bust’ event in the study region [39]. We pooled the numbers of photographs of each predator species from these cameras each month at each site (see [39] for details and electronic supplementary material, table S1).

### 2.4. Spinifex cover and seed surveys

To measure the cover of the dominant vegetation (spinifex, *T. basedowii*), we visually scored the percentage cover in a 2.5 m radius around six traps at each trapping grid that was used to sample small vertebrates. In addition, a seed index (0–5, where 0 represents no seeding present and 5 represents every plant bearing maximum seed) was used to score spinifex seed production. Spinifex seed was chosen due to its dominance in the landscape and because it is an important component of the diets of both main species of study rodent, *N. alexis* and *P. hermannsburgensis*, representing up to 52% of their diet by occurrence [40]. Cover and seed estimates were averaged per trap and then for each trapping grid within each site, per trip, over 17–22 years for each of the nine sites as described in Greenville [38] (see electronic supplementary material, table S1).

### 2.5. Characterizing rainfall and wildfire

Daily rainfall data from automatic weather stations (Environdata, Warwick, Queensland, Australia) located at each site were used to calculate rainfall variables: total rainfall for each trapping month, number of rain days, mean rainfall per day, mean and maximum event size and rainfall lags for each month up to a year (electronic supplementary material, table S1). Cumulative rainfall in the previous two months up to that in the previous 12 months was also calculated. Rainfall variables identified as important predictors for mammals and reptiles are number of rain days in a month and the mean rainfall-event size lagged two trips prior [38]. Rainfall generally falls from October to April, but is highly unpredictable [22]. For spinifex cover and seeding, eight-month cumulative rainfall was used to represent this rain period and the attendant variability.

Large-scale wildfires (greater than 1000 km^2^) have occurred three times (1974, 2001/2002 and 2011/2012) in the study region since 1972 and the mean wildfire return interval for the region is 26 years [21,41]. To investigate if wildfire had an effect on vertebrate captures, the time (years) since last wildfire was calculated for each trapping grid based on remotely sensed mapping of fire scars [21,41].

### 2.6. Structural equation modelling

**Figure 2.**
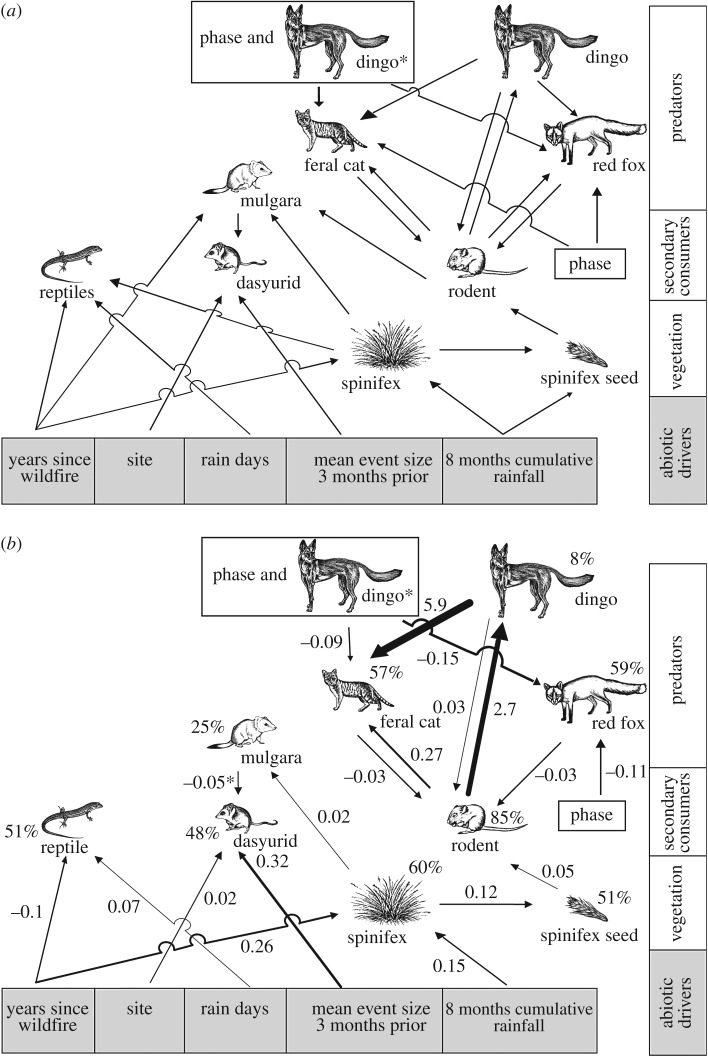
Structural equation interaction model. (*a*) Conceptual model describing proposed interactions between abiotic drivers, species, species groups and trophic levels predicted under the pulse-reserve paradigm and knowledge of the factors influencing the abundance and spatial dynamics of Australian desert systems. (*b*) Results from SEM for the same model quantifying significant interactions. Values above the drawings are percentage deviances explained. Standardized path coefficients are shown for each arrow, and arrow thickness is proportional to effect size and represents significant path coefficients (*p *< 0.05). Non-significant interactions are not shown. Artwork by A. Foster. *Interaction term between dingo numbers and population phase of rodent prey.

To investigate the species interactions and processes in our system, we built a phenomenological model using a graph-theoretic approach to SEM. Each species or species group was entered as a node and the direction of the arrow represented a hypothesised causal pathway or direct interaction (figure 2*a*). This approach is not based on a global covariance matrix; rather, each node is modelled separately, allowing different statistical techniques to be used for local estimation [42]. Thus, we derived nine separate models, totalling 26 direct interactions or processes (table 1; electronic supplementary material, table S1). We investigated the effect of the predictor variables on small mammal and reptile captures by using a Poisson generalized linear mixed model (GLMM), with an offset for the number of trapping nights to account for unequal trapping effort (total sampling effort pooled across all nine sites for each model: rodents: *n* = 103; dasyurids: *n *= 1118; mulgara: *n* = 1386, reptiles: *n *= 911; table 1; electronic supplementary material, S1). Because the same trapping grid or remote camera was sampled each trip, it (trip) was added as the random factor to account for observations repeated over time [43]. We modelled the effects of the predictor variables on spinifex cover and seed as proportional odds, and a binomial GLMM was used (total sampling effort pooled across all nine sites for each model: *n* = 774 and 773, respectively; table 1; electronic supplementary material, S1). Lastly, we modelled 2 years of predator data from remote camera traps using a Poisson GLMM, with the number of nights each camera was operational used as an offset to account for camera malfunctions (total sampling effort pooled across all nine sites for each model: *n *= 114; table 1; electronic supplementary material, S1). Pitfall captures or numbers of photographs of mulgara, feral cat, red fox, dingo and rodents entered as predictor variables were standardized for sampling effort (table 1). Because only 2 years of feral cat, red fox and dingo data were available, only the corresponding 2 years of rodent pitfall trapping could be used in this submodel.

**Table 1.** Individual models for each node in the structural equation model. Trip was entered as a random factor to account for the repeated measures. Mean rainfall event size2 = mean rainfall event size two trips prior. Predictor variables from captures or number of photographs of mulgara, feral cat, red fox, dingo and rodents entered as predictor variables were standardized for sampling effort. Offsets for total trapping nights and camera days were added to account for unequal sampling effort. Data from nine sites across 2–22 years. *Predictor variables *z*-transformed to allow model convergence.

model	no. years
insectivorous dasyurids ∼ Poisson	
log(dasy) ← site + mean rainfall event size2 + mulgara/100 trap nights + (approx. 1|Trip) + offset(trap nights) + *ε*	17–22
spinifex cover ∼ binomial	
logit(spinifex cover) ← years since wildfire + 8 month cumulative rainfall + (approx. 1|trip) + *ε*	17–22
spinifex seed ∼ binomial	
logit(spinifex seed) ← spinifex cover + 8 month cumulative rainfall + (approx. 1|trip) + *ε*	17–22
rodents ∼ Poisson	
log(rodents) ← spinifex seed + feral cat/camera night + red fox/camera night + dingo/ camera night + (approx. 1|trip) + offset(trap nights) + *ε*	2
mulgara ∼ Poisson	
log(mulgara) ← rodents/100 trap nights + spinifex cover + years since wildfire + (approx. 1|trip) + offset(trap nights) + *ε*	17–22
feral cat ∼ Poisson	
log(feral cat) ← rodents/100 trap nights + dingo/camera night + phase + phase × dingo/camera night + (approx. 1|Trip) + offset(camera nights) + *ε*	2
red fox* ∼ Poisson	
log(red fox) ← rodents/100 trap nights + dingo/camera night+ phase + phase × dingo/camera night + (approx. 1|trip) + offset(camera nights) + *ε*	2
dingo ∼ Poisson	
log(dingo) ← rodents/100 trap nights + (approx. 1|trip) + offset(camera nights) + *ε*	2
reptiles ∼ Poisson	
log(reptiles) ← years since wildfire + spinifex cover + rain days + (approx. 1|trip) + offset(trap nights) + ε	17–22

Previous research in the study region has found that the strength of top predator suppression on the two species of mesopredator is influenced by the population phase of their shared rodent prey [39]. Therefore, we entered population phase (boom, decline and bust; coded as 1, 2, 3) and the interaction between dingoes and population phase (phase) into the model following the approach and findings of Greenville *et al*. [39]. We calculated standardized coefficients for each pathway by computing the change in the response variable from its predicted minimum value to its predicted maximum value, adjusted for the response's maximum range [44]. All analyses were performed in R v. 2.15.3 [45], using the package lm4 [46]. Owing to the various dataset lengths and the inclusion of mixed effects, global model fits for a piecewise SEM could not be calculated. Instead, we determined the relative fit of each local model by calculating the percentage deviance explained (deviance for the null model minus the deviance of the fitted model, divided by the deviance of the null model) [47], an approach that has been used successfully in past studies using SEMs [48].

### 2.7. Model justification

The proposed causal pathways between variables in the conceptual model (figure 2*a*) were determined by our *a priori* knowledge of predator–prey dynamics, the pulse-reserve paradigm and prior knowledge of the factors influencing the abundance and spatial dynamics of Australian desert systems. Both bottom-up and top-down effects can operate in arid systems [39,49,50] and thus are incorporated into the model. We chose rainfall, wildfire and spatial driver variables based on our prior knowledge of Australian desert systems (e.g. [17,38,51–54]). Captures across sites were pooled for rodents, the small carnivorous mulgara (*D. blythi*), and reptiles, as spatial population synchrony is exhibited for these groups, but not for smaller, insectivorous dasyurid marsupials [38,53]. Under the pulse-reserve paradigm, it was hypothesized that changes in spinifex cover would influence spinifex seed production, as larger plants (greater cover) could compete more successfully for resources and produce more seeds or have more successful seeding events. Rodents consume large amounts of spinifex seed, and seeding events are positively associated with population increases [40,55]. Even though Australian rodents consume higher levels of invertebrates than in other desert regions, invertebrate prey show a variable response to rainfall and thus we hypothesize that spinifex seed production is the most important factor influencing population irruptions [56,57]. Increases in rodent populations lead in turn to increases in predator populations, but predation pressure on rodents may differ between native and non-native predators [30,31]. For example, Greenville *et al*. [53] found no predatory effects of the native mulgara on rodent populations, but feral cats and the introduced red fox can force populations to extinction across Australia [58]. In addition, rodents can form an important component in the diet of the dingo, but the number of rodents consumed per day was highest for the introduced cat and fox [30,59]. We did not include a pathway between the feral cat and introduced red fox, as previous research suggested that there was no interaction between these two species in our study system [39]. Reptile populations can be influenced by spinifex cover, wildfire and number of rain days [38,54,60,61]. Populations of small dasyurids differ across space and can be influenced by both bottom-up effects from rainfall and top-down effects from the predatory mulgara [38,62,63]. Wildfire, spinifex cover and rodent captures (prey) are important drivers for mulgara populations [64–66].

### 2.8. Changes in rainfall and wildfire from climate change

To determine how the two key drivers, rainfall and wildfire, may influence interactions and processes between trophic groups under a changing climate, we simulated projected changes in years since wildfire and cumulative eight-month rainfall 100 years into the future. We generated a current rainfall scenario for 2000 samples from the negative binomial distribution, using parameters estimated from actual eight-month cumulative rainfall data measured by on-site weather stations (*µ* = 152, size = 1.57, *n* = 2000). Extreme rainfall events (greater than 95th quantile) have increased in frequency and magnitude over the last approximately 100 years in the Simpson Desert, leading to an approximate increase of 4 mm per year on average [24]. Thus, we generated eight-month cumulative rainfall datasets using a negative binomial distribution to project an increase in rainfall for successive 12-year intervals (12 time steps) over the next 100 years (100 years: *µ* = 419, *n* = 2000; see electronic supplementary material, table S2). The negative binomial distribution was chosen over the Poisson as rainfall in arid environments is over-dispersed due to large numbers of nil or small rainfall events per year. Histograms were used to confirm that simulated datasets had similar distributions to actual rainfall datasets (electronic supplementary material, figure S1). Changes in future rainfall are uncertain, but our rainfall parameters are all within projected changes for central Australia, mainly due to expected increases in extreme rainfall events [67,68].

Wildfire return intervals are predicted to decrease due to changes in rainfall patterns and evaporation rates in central Australia [24,25]. To simulate a reduction in the years since wildfire across the next 100 years, we used a negative binomial distribution and generated a current wildfire scenario based on parameter estimates from the actual wildfire data (*µ* = 21, size = 3.34, *n* = 2000). We assumed that in 100 years, the mean time between wildfires would change from 21 to 10 years (*µ* = 10, size = 3.34, *n* = 2000) and generated 12 time steps over the next 100 years (see electronic supplementary material, table S2). All mean years since wildfire parameters used are within reported fire return intervals for central Australia [23,69]. As for rainfall, we used histograms to confirm that simulated datasets had similar distributions to those for actual wildfires (electronic supplementary material, figure S2).

The simulated datasets were entered into the fixed SEM equations (table 1) using the standardized path coefficients (figure 2), and predicted values for each trophic level were re-entered at each node from spinifex cover, then spinifex seed and lastly the rodent node. In addition, to test the influence of top-down effects from introduced predators, we constructed three scenarios: firstly, with all mammalian predators using existing predator datasets; secondly, without the introduced red fox and feral cat; and thirdly, without any mammalian predators. Models were run 2000 times, except for the rodent models where predator datasets were smaller due to predator monitoring using remote camera traps running for only 2 years (*n* = 342; electronic supplementary material, table S1).

## 3. Results

### 3.1. Structural equation modelling

From a total of 26 direct interactions and processes proposed in our *a priori* model (figure 2*a*), 19 were significant (figure 2*b*; electronic supplementary material, table S3). Spinifex cover was positively associated with increases in eight-month cumulative rainfall and the spinifex seed index increased with increasing cover (figure 2*b*; electronic supplementary material, table S3). Rodent captures increased with increases in the spinifex seed index and were associated negatively with the introduced red fox and feral cat but positively with the native dingo (figure 2*b*; electronic supplementary material, table S3). Insectivorous dasyurids were influenced by both spatial and bottom-up effects from the mean rainfall event size two months prior, but also negatively associated with top-down effects from the predatory mulgara (figure 2*b*; electronic supplementary material, table S3). As predicted from our *a priori* model, the mulgara was associated positively with spinifex cover. Reptile captures were associated positively with the number of rain days and negatively with years since wildfire (figure 2*b*; electronic supplementary material, table S3). Seven pathways in our *a priori* model (figure 2*a*) were not significant; spinifex cover was not associated with reptile captures, mulgara captures were not associated with rodent numbers or year since wildfire, and the spinifex seed index was not associated with eight-month cumulative rainfall. Prey population phase was not associated with feral cat abundance, and there was no direct effect of dingoes on red foxes (figure 2*b*; electronic supplementary material, table S3). There was a positive effect of dingoes on feral cats, but the interaction of phase and dingo was negative on both feral cats and foxes (figure 2*b*; electronic supplementary material, table S3). Feral cat numbers increased with rodent captures, but red foxes did not (figure 2*b*; electronic supplementary material, table S3).

### 3.2. Climate change

**Figure 3.**
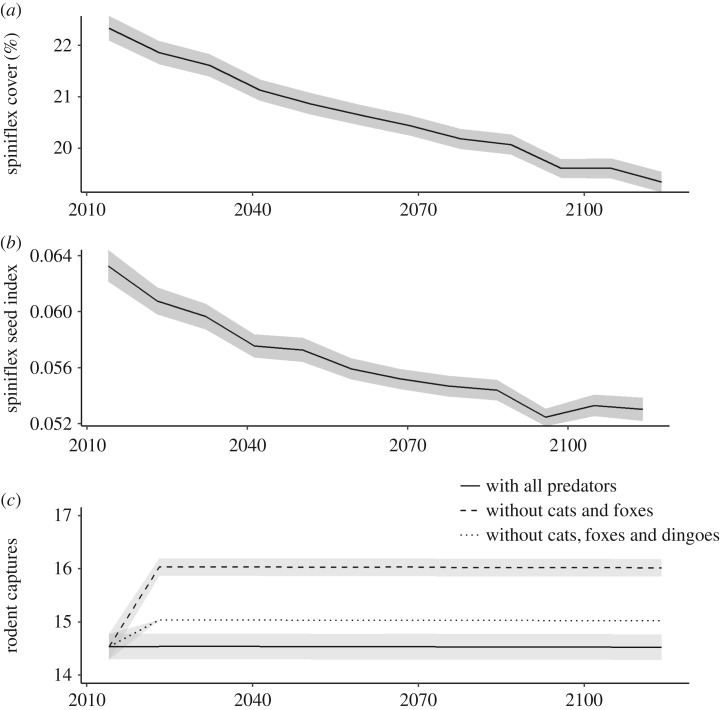
Response from simulated changes in rainfall and wildfire as a result of climate change over the next 100 years. Projected results (±s.e.) for changes in (*a*) percentage spinifex cover, (*b*) spinifex seed index and (*c*) rodent captures (standardized per 100 trap-nights per 1 ha grid) with all mammalian predators present, introduced predators (cats and foxes) removed and all mammalian predators removed, under a changing rainfall and wildfire scenario.

There were decreases in projected spinifex cover (figure 3*a*) and the spinifex seed index (figure 3*b*) over the next 100 years, but not in the mean projected rodent captures from changes in rainfall and wildfire patterns (figure 3*c*). When introduced predators were removed from the models, there was an increase in predicted rodent captures of approximately 9%. However, when both native and non-native mammalian predators were removed, rodent captures were projected to increase by approximately 3% (figure 3*c*).

## 4. Discussion

As predicted from our *a priori* model based on the pulse-reserve paradigm, higher rainfall resulted in significantly increased captures of small mammals via changes in food resources (seeds) and also led to increased captures of reptiles. As expected, the dominant vegetation cover, spinifex, increased with rainfall and with years since wildfire. While spinifex seed production also increased predictably with the cover of the parent plant, increases in the cover of spinifex provide fuel for wildfires [23] which can in turn indirectly influence populations of small mammals through changes in food availability. However, these flow-on effects differed between rodents and dasyurids. As predicted, native rodents appeared to be limited by top-down effects from introduced predators (feral cats and red foxes), whereas populations of small dasyurids exhibited a response to the native mulgara, a larger dasyurid predator.

The interaction between the top predator, the dingo and the population phase of its prey had a negative effect on the introduced mesopredators, the red fox and feral cat. This suggests that the strength of top-down effects on the mesopredators was strongest in the ‘bust’ and ‘decline’ phases of their shared rodent prey [39]. The positive association between dingoes and feral cats may reflect simultaneous increases in both populations during ‘boom’ conditions driven by rainfall-led productivity pulses. Conversely, there was no direct association between dingoes and red foxes. This suggests that foxes have a longer lag-time in responding to prey populations, perhaps because they are not always present in the desert system and migrate in from more mesic areas around the study region post-rain [70,71]. Further support for the slower response due to migration can be found in the negative association between phase and red foxes. During the ‘bust’ phase of rodent prey, red foxes can become undetectable, while dingoes and feral cats can persist in low numbers [39]. Red fox numbers were not influenced by rodent captures, suggesting that the red fox has a more flexible diet than the feral cat [72].

There was no direct relationship between spinifex seed productivity and cumulative rainfall, but the results suggest that rainfall indirectly influenced seed production through the growth of spinifex hummocks. As a group, reptiles were influenced by wildfire and number of rain days, but surprisingly not by spinifex cover, even though spinifex can be completely removed by wildfire. The grouping of reptiles above the species level may have masked more subtle processes and differences in responses by individual species, especially as these responses can be divergent [60,73]. Alternatively, Greenville *et al*. [54] found no relationship between spinifex cover and abundance of six species of reptiles from the same region, but did find wildfire important for driving the spatial dynamics for over half of the species studied. Thus, we suggest that wildfire and other processes, such as predation or food [74], may be more important than the level of spinifex cover *per se*.

### 4.1. Climate change

Consistent with our prediction, an increase in vegetation cover stimulated by the higher rainfall projected for central Australia was offset by increases in wildfire frequency, leading to a predicted decrease in the dominant plant cover, spinifex, over the next 100 years. Such a decrease would suggest a shift to smaller and younger spinifex plants and, in turn, a reduction in reproductive output, thus illustrating that the interaction between abiotic drivers and vegetation can have complex effects. Abrupt climate change can lead to rapid shifts in the vegetation community. For example, both charcoal and pollen samples show that sudden changes in climate can increase fire activity and swiftly drive shifts in vegetation structure and composition [75,76]. Using simulations to predict vegetation change from anthropogenic climate shifts, Mouillot *et al*. [77] found that climate change decreased the time between wildfires and placed vegetation on a trajectory from wooded towards shrub-dominated landscapes. After wildfire in spinifex grasslands, there is an increase in cover of annual and perennial vegetation [23]. Given that spinifex grasslands occupy over a quarter of the area of continental Australia (figure 1; [69]), changes in rainfall and fire frequency may lead to a large-scale shift in the cover of the dominant vegetation and increases in annual and perennial species across central and northern arid regions of Australia, further contributing to global vegetation alteration.

Contrarily, rodent abundance was not predicted to change along with decreases in spinifex seeding under the modelled climate change scenario. The weak link between spinifex seed and rodents could have four possible causes. Firstly, rodents may be able to supplement their food with seed from other plant species, or invertebrates. For example, Murray & Dickman [40] found some dietary switching, especially in the Austral autumn, when invertebrates could make up to 60% of the diets of some desert rodents. Secondly, during resource pulses, the increase in spinifex seed may be in excess of what is required for rodent populations to irrupt. For example, rodent populations in central Australia respond when a rainfall threshold is reached and, beyond this, there is likely to be an excess of food [24,78]. However, given their fast reproductive rates and rapid population responses to rainfall events, rodents could be expected to maximize their response to the available food supply. In addition, rapid population declines after resource pulses occur in desert systems, suggesting that competition for food may be operating [79,80]. Thirdly, the spinifex seed index may be a coarse measure of seed output; spinifex can undergo infrequent mast seeding events, resulting in weak links between spinifex cover and seed output [81]. Lastly, rodent populations may be limited by predation from introduced predators. Introduced predators often depredate rodents and other native mammals more heavily than do native predators [30,31] and are one of the key drivers of extinction of Australian mammals [82]. Thus, when mammalian predators were removed from our model, there was an increase in the mean predicted captures of rodents that was most pronounced when only the introduced predators were removed. Although the dingo will eat rodents, this native species prefers to hunt other, larger prey, than the introduced fox and cat [30,72,83]. The ostensibly paradoxical result that the increase in rodent captures is lower with no predators than it is when dingoes are present (figure 3*c*) may reflect two possibilities: it could be an artefact of the model that arose from a transitory but unrepresentative positive effect of dingoes on rodents during the limited 2-year timeframe of the camera deployment. This seems unlikely, as the potential positive effects of dingoes on rodents have been documented broadly in other desert studies [84,85]. More likely, the model result reflects compensatory survival or reproductive responses by rodents, with animals that remain after limited off-take by dingoes experiencing reduced social pressure and prolonged reproduction [86]. Whatever the case, it is clear that predation from introduced species limits prey populations compared with changing abiotic conditions from climate change. Introduced predators quickly exploit the open areas created after wildfire [87], and the projected decrease in vegetation cover from climate change may provide a negative feedback loop that allows increased predation from introduced predators on rodents and other native prey.

The SEM framework made it possible to integrate multiple long-term datasets from our studies in central Australia and to make predictions about the effects of direct and indirect interactions among existing species [42,48,88]. This is the first time this has been possible using such an extensive empirical dataset on a highly diverse biotic assemblage. However, we suggest that the framework can be enhanced still further. Firstly, the SEM framework did not account for novel interactions. For example, invasive species such as the European rabbit (*Oryctolagus cuniculus*) and house mouse (*Mus musculus*) are largely absent in our study region, but increases in the magnitude and frequency of large (greater than 90th quantile) rainfall events may allow these species to establish [22,24]. The influence of these introduced species on vegetation and predator populations should be the focus of future research. Secondly, weak interactions may not be well represented in our model, but they can have important effects on the overall stability of food webs [7,89]. Thirdly, the sensitively of the model predictions could be tested by running other climate change scenarios not considered here. Lastly, little is known about other important components of the community, such as invertebrates, birds and interactions between reptile, dasyurid and eutherian predators (e.g. [90–92]), or processes such as nutrient cycling. For example, interactions between introduced predators and small native dasyurid predators are largely unstudied and hard to predict. We suggest that these areas be foci for further research.

## 5. Conclusion

The population responses among the diverse desert-dwelling biota that we studied may be influenced by both bottom-up and top-down drivers as predicted by the pulse-reserve paradigm. For mammal populations, which have been depleted more than any other component of Australia's diverse inland vertebrate assemblages [22], our results indicate that declines in a key food and shelter resource—spinifex—are of far less import than the impacts of introduced predators under future climate change scenarios. Although our SEM models did not account for all possible interactions as they brought together *a priori* knowledge about individual species and systems, our analyses suggest that these models can yield novel and powerful insights into the functioning of diverse ecological systems. For our system in particular, we found that while vegetation cover can be expected to decline due to climate change, the primary influence on prey populations is top-down suppression from introduced predators.

## Supplementary Material

Figure S1

## Supplementary Material

Figure S2

## Supplementary Material

Table S1

## Supplementary Material

Table S2

## Supplementary Material

Table S3

## Data accessibility

Long-term datasets containing small vertebrate and vegetation data to construct the structural equation model can be derived from data at LTERN [93–99], or http://aekos.org.au/collection/sydney.edu.au/DERG [100] and remote camera predator data can be accessed from AEKOS [101].
