# Supplementary material for: Desert mammal populations are limited by introduced predators rather than future climate change
Source: R Soc Open Sci. 2017 Nov 1;4(11):170384. doi: 10.1098/rsos.170384 (PMC5717625; doi:10.1098/rsos.170384)
Supplement: Figure S1 [file rsos170384supp1.docx]

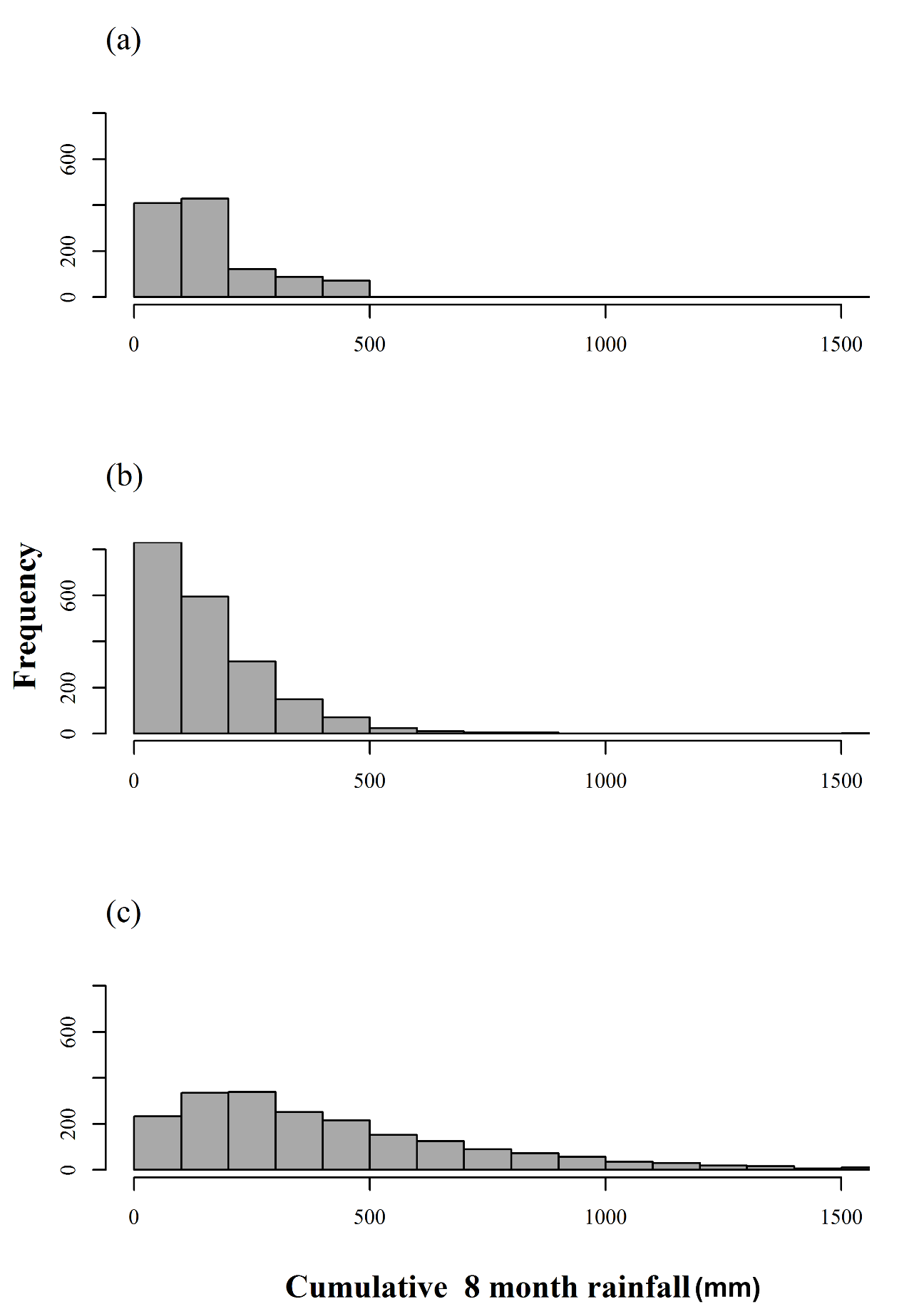


Figure S1: Eight month cumulative rainfall (mm) data for (a) actual dataset, (b) simulated dataset for current rainfall (*n* = 2000), and (c) simulated dataset for rainfall in 100 years. See Table S1 for parameters used to simulate data from a negative binomial distribution.
