# Supplementary material for: Desert mammal populations are limited by introduced predators rather than future climate change
Source: R Soc Open Sci. 2017 Nov 1;4(11):170384. doi: 10.1098/rsos.170384 (PMC5717625; doi:10.1098/rsos.170384)
Supplement: Figure S2 [file rsos170384supp2.docx]

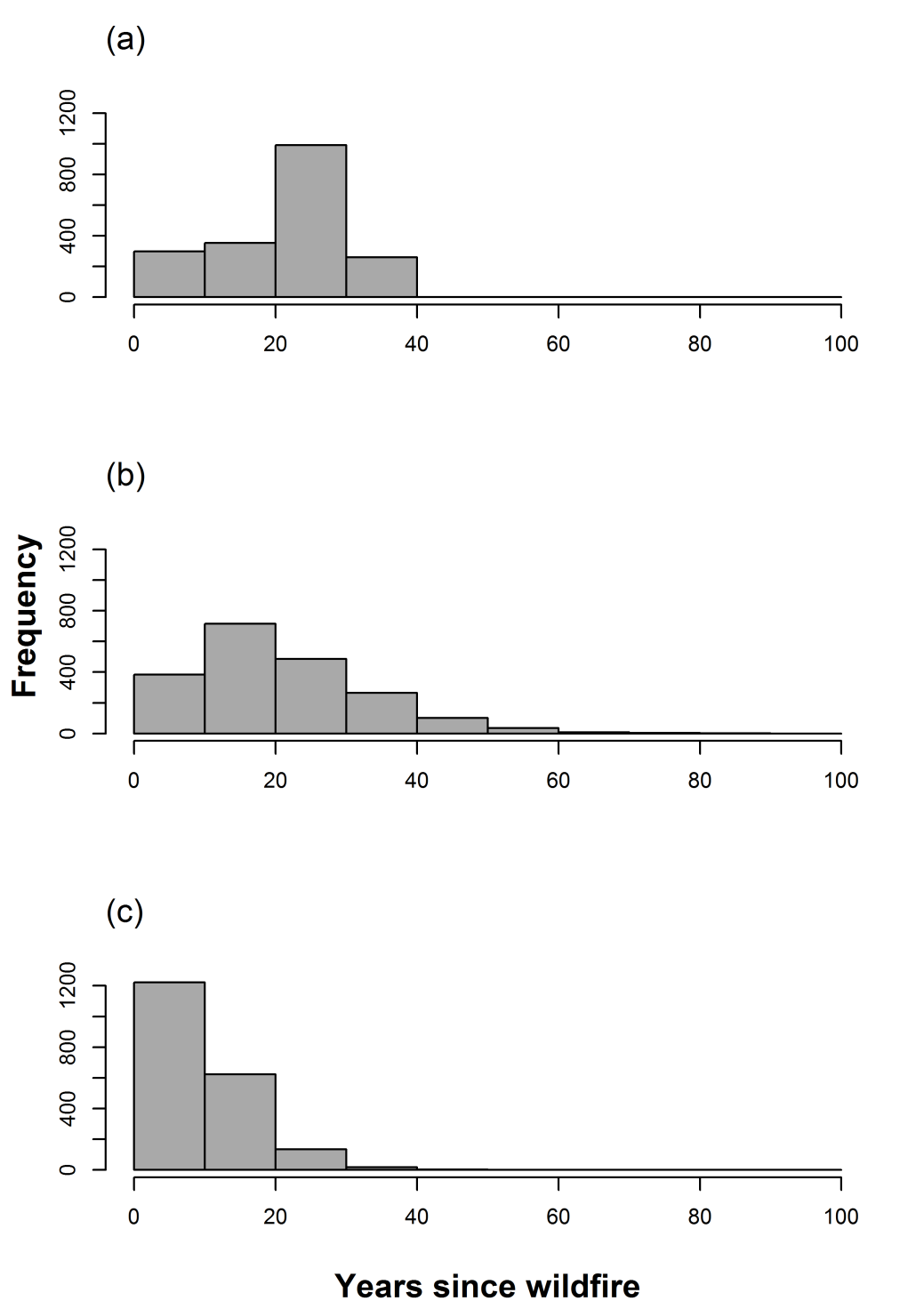


Figure S2: Years since wildfire for data for (a) actual dataset, (b) simulated dataset for current rainfall (*n* = 2000), and (c) simulated dataset for wildfire in 100 years. See Table S1 for parameters used to simulate data from a negative binomial distribution.
