# Supplementary material for: Desert mammal populations are limited by introduced predators rather than future climate change
Source: R Soc Open Sci. 2017 Nov 1;4(11):170384. doi: 10.1098/rsos.170384 (PMC5717625; doi:10.1098/rsos.170384)
Supplement: Table S1 [file rsos170384supp3.docx]

Table S1: Description of the variables used in the structural equation models. The distribution is given for response variables only. Variables used follow Greenville [1], Greenville [2], and Greenville [3].

| Variable | Type | Distribution | Notes |
| --- | --- | --- | --- |
| Dasyurid | Abundance count; 0-max | Poisson | Nine sites, <=23 years |
| Reptiles | Abundance count; 0-max | Poisson | Nine sites, <=23 years |
| Mulgara | Abundance count; 0-max | Poisson | Nine sites, <=23 years |
| Rodents | Abundance count; 0-max | Poisson | Nine sites, <=23 years |
| Cat | Abundance count; 0-max | Poisson | 2 years, pooled by sites |
| Fox | Abundance count; 0-max | Poisson | 2 years, pooled by sites |
| Dingo | Abundance count; 0-max | Poisson | 2 years, pooled by sites |
| Spinifex cover | 0-max% | Binomial | Nine sites, <=23 years |
| Spinifex seed | Index; 0,1,2,3,4,5 | Binomial | Nine sites, <=23 years |
| Site | Categorical; 1-9 |  | Nine sites, <=23 years |
| Mean event size 2 months prior | Continuous |  | Nine sites, <=23 years |
| 8 months cumulative rainfall | Continuous |  | Nine sites, <=23 years |
| Rain days | Continuous |  | Nine sites, <=23 years |
| Years since wildfire | Continuous, integer |  | Greenville [2] and NAFI [4] |

**References:**

1. Greenville A.C. 2015 The role of ecological interactions: how intrinsic and extrinsic factors shape the spatio-temporal dynamics of populations [PhD Thesis]. Sydney, University of Sydney.

2. Greenville A.C., Dickman C.R., Wardle G.M., Letnic M. 2009 The fire history of an arid grassland: the influence of antecedent rainfall and ENSO. *Int J Wildland Fire* **18**, 631-639. (doi:10.1071/WF08093).

3. Greenville A.C., Wardle G.M., Tamayo B., Dickman C.R. 2014 Bottom-up and top-down processes interact to modify intraguild interactions in resource-pulse environments. *Oecologia* **175**, 1349-1358. (doi:10.1007/s00442-014-2977-8).

4. NAFI. 2013 North Australian Fire Information. <http://www.firenorth.org.au/nafi2/>.
