# Supplementary material for: Desert mammal populations are limited by introduced predators rather than future climate change
Source: R Soc Open Sci. 2017 Nov 1;4(11):170384. doi: 10.1098/rsos.170384 (PMC5717625; doi:10.1098/rsos.170384)
Supplement: Table S2 [file rsos170384supp4.docx]

Table S2: Parameter µ (mean) used to simulate the change in wildfire and rainfall (mm) due to climate change over 100 years, using 12 time steps. Simulated values were generated from a negative binominal distribution (rainfall: size (dispersion) = 1.57, *n* = 2000, and wildfire: size = 3.34, *n* = 2000; size was estimated from actual datasets for both rainfall and wildfire, as was µ for 2014).

| Time step | Time since wildfire (mu) | 8 months cumulative rainfall (mu) |
| --- | --- | --- |
| 2014 | 21 | 152 |
| 2023 | 20 | 176.27 |
| 2032 | 19 | 200.54 |
| 2041 | 18 | 224.81 |
| 2050 | 17 | 249.08 |
| 2059 | 16 | 273.35 |
| 2068 | 15 | 297.62 |
| 2077 | 14 | 321.89 |
| 2086 | 13 | 346.16 |
| 2095 | 12 | 370.43 |
| 2104 | 11 | 394.7 |
| 2114 | 10 | 418.97 |
