# Supplementary material for: Desert mammal populations are limited by introduced predators rather than future climate change
Source: R Soc Open Sci. 2017 Nov 1;4(11):170384. doi: 10.1098/rsos.170384 (PMC5717625; doi:10.1098/rsos.170384)
Supplement: Table S3 [file rsos170384supp5.docx]

Table S3: Generalised linear mixed model results for each local model in the piecewise structural equation model. Fixed effects shown with unstandardized estimates. See methods for model details. *Predictor variables z-transformed to allow model convergence.

| **Variable** | **Estimate** | **SE** | **Z-value** | ***P*-value** |
| --- | --- | --- | --- | --- |
| **Dasyurids** |  |  |  |  |
| Intercept | 0.31 | 0.11 | 2.80 | 0.005 |
| Site | −0.04 | 0.02 | −2.05 | 0.04 |
| Mean rainfall event size 2 months prior | 0.009 | 0.001 | 7.95 | *P*<0.01 |
| Mulgara | −0.21 | 0.04 | −5.27 | *P*<0.01 |
|  |  |  |  |  |
| **Spinifex Cover** |  |  |  |  |
| Intercept | −2.48 | 0.08 | −31.27 | *P*<0.01 |
| Years since wildfire | 0.04 | 0.001 | 33.09 | *P*<0.01 |
| 8 months cumulative rainfall | 0.001 | 0.0002 | 7.94 | *P*<0.01 |
|  |  |  |  |  |
| **Spinifex seed** |  |  |  |  |
| Intercept | −5.52 | 0.54 | −10.14 | *P*<0.01 |
| 8 months cumulative rainfall | 0.0005 | 0.0009 | 0.53 | 0.594 |
| Spinifex cover | 0.046 | 0.009 | 5.12 | *P*<0.01 |
|  |  |  |  |  |
| **Rodents** |  |  |  |  |
| Intercept | 2.70 | 0.42 | 6.39 | *P*<0.01 |
| Spinifex seed | 0.11 | 0.02 | 6.03 | *P*<0.01 |
| Dingo | 0.85 | 0.18 | 4.71 | *P*<0.01 |
| Red fox | −2.95 | 0.49 | −5.97 | *P*<0.01 |
| Feral cat | −1.22 | 0.37 | −3.28 | *P*<0.01 |
|  |  |  |  |  |
| **Mulgara** |  |  |  |  |
| Intercept | −2.29 | 0.23 | −10.02 | *P*<0.01 |
| Rodents | 0.002 | 0.003 | 0.74 | 0.45906 |
| Spinifex cover | 0.013 | 0.005 | 2.85 | *P*<0.01 |
| Years since wildfire | 0.009 | 0.006 | 1.54 | 0.12334 |
|  |  |  |  |  |
| **Feral cat** |  |  |  |  |
| Intercept | −1.31 | 0.61 | −2.17 | 0.0. |
| Rodents | 0.008 | 0.003 | 2.54 | 0.01 |
| Dingoes | 12.93 | 2.18 | 5.94 | *P*<0.01 |
| Phase | 0.19 | 0.16 | 1.20 | 0.23 |
| Dingo × Phase | −2.02 | 0.89 | −2.28 | 0.02 |
|  |  |  |  |  |
| **Red fox*** |  |  |  |  |
| Intercept | −1.23 | 0.49 | −2.48 | 0.01 |
| Rodents | 0.07 | 0.06 | 1.15 | 0.25 |
| Dingoes | −0.56 | 0.39 | −1.42 | 0.15 |
| Phase | −1.32 | 0.50 | −2.67 | 0.01 |
| Dingo × Phase | −2.14 | 0.35 | −6.20 | *P*<0.01 |
|  |  |  |  |  |
| **Dingoes** |  |  |  |  |
| Intercept | −0.12 | 0.66 | −0.18 | 0.86 |
| Rodents | 0.02 | 0.003 | 6.17 | *P*<0.01 |
|  |  |  |  |  |
| **Reptiles** |  |  |  |  |
| Intercept | 2.13 | 0.08 | 25.17 | *P*<0.01 |
| Rain days | 0.03 | 0.008 | 3.81 | *P*<0.01 |
| Spinifex cover | 0.0006 | 0.0013 | 0.48 | 0.62934 |
| Years since wildfire | −0.006 | 0.002 | −4.18 | *P*<0.01 |
